# The tyrosine 73 and serine 83 dephosphorylation of H1N1 swine influenza virus NS1 protein attenuates virus replication and induces high levels of beta interferon

**DOI:** 10.1186/s12985-019-1255-0

**Published:** 2019-12-05

**Authors:** Jinghua Cheng, Jie Tao, Benqiang Li, Ying Shi, Huili Liu

**Affiliations:** 1 Institute of Animal Science and Veterinary Medicine, Shanghai, Academy of Agricultural Science, Shanghai, 201106 China; 2 Shanghai Key Laboratory of Agricultural Genetic Breeding, Shanghai, 201106 China; 3 Shanghai Engineering Research Center of Pig Breeding, Shanghai, 201302 China

**Keywords:** Swine influenza virus, NS1 protein, Phosphorylation, Interferon responses, RIG-I

## Abstract

**Background:**

Nonstructural protein 1 (NS1) is a virulence factor encoded by influenza A virus (IAV) that is expressed in the nucleus and cytoplasm of host cells during the earliest stages of infection. NS1 is a multifunctional protein that plays an important role in virus replication, virulence and inhibition of the host antiviral immune response. However, to date, the phosphorylation sites of NS1 have not been identified, and the relationship between phosphorylation and protein function has not been thoroughly elucidated.

**Method:**

In this study, potential phosphorylation sites in the swine influenza virus (SIV) NS1 protein were bioinformatically predicted and determined by Phos-tag SDS-PAGE analysis. To study the role of NS1 phosphorylation sites, we rescued NS1 mutants (Y73F and S83A) of A/swine/Shanghai/3/2014(H1N1) strain and compared their replication ability, cytokine production as well as the intracellular localization in cultured cells. Additionally, we used small interfering RNA (siRNA) assay to explore whether changes in the type I IFN response with dephosphorylation at positions 73 and 83 were mediated by the RIG-I pathway.

**Results:**

We checked 18 predicted sites in 30 SIV NS1 genes to exclude strain-specific sites, covering H1N1, H1N2 and H3N2 subtypes and identified two phosphorylation sites Y73 and S83 in the H1N1 SIV protein by Phos-tag SDS-PAGE analysis. We found that dephosphorylation at positions 73 and 83 of the NS1 protein attenuated virus replication and reduced the ability of NS1 to antagonize IFN-β expression but had no effect on nuclear localization. Knockdown of RIG-I dramatically impaired the induction of IFN-β and ISG56 in NS1 Y73F or S83A mutant-infected cells, indicating that RIG-I plays a role in the IFN-β response upon rSIV NS1 Y73F and rSIV NS1 S83A infection.

**Conclusion:**

We first identified two functional phosphorylation sites in the H1N1 SIV protein: Y73 and S83. We found that dephosphorylation at positions 73 and 83 of the NS1 protein affected the antiviral state in the host cells, partly through the RIG-I pathway.

## Introduction

Swine influenza (SI) is a highly contagious respiratory disease that is characterized by fever, weight loss and acute respiratory problems. It is caused by influenza A virus (IAV), which belongs to the Orthomyxoviridae family. Outbreaks of swine flu cause significant morbidity and growth retardation in pigs, leading to a considerable economic loss to the infected farms [1, 2]. The major strains found in swine herds are the H1N1, H1N2 and H3N2 subtypes. Because of their broad susceptibility, pigs are important hosts and are considered “mixing vessels” that can foster the generation of novel reassortant influenza viruses [3]. With the H1N1 pandemic of 2009 as example, it is thought to be the swine-origin virus that spread globally [4]. Pathogenicity of influenza viruses is determined by many factors, so it is necessary to understand the pathogenic mechanisms of this virus for disease control [5].

IAV possesses a segmented genome with eight single stranded negative-sense RNA molecules. The surface glycoproteins hemagglutinin (HA) and neuraminidase (NA) are the key antigens and can be used to classify IAV into different subtypes [6]. Nonstructural protein (NS1), which is the product of the smallest RNA segment, is a virulence factor of the influenza virus. Hale et al. reviewed the multifunctional NS1 protein of IAV in their paper [7]. The most important biological functions of the NS1 protein are its antagonism of the host innate response and its promotion of the effective replication of the virus [8]. Wang et al. reported that a recombinant IAV lacking the NS1 gene only replicates efficiently in type I interferon (IFN)-α/ -deficient systems [9]. Influenza viruses lacking the NS1 protein or NS1-truncated mutants have been proven induced higher levels of cytokines, attenuated and immunogenic in mice and in pigs [5, 10]. Additionally, some key amino acid sites have also been identified to affect virus growth as well as the induction of type I IFN in vitro and in vivo. For example, Jiao et al. reported that the NS1 protein is critical for the pathogenicity of H5N1 influenza viruses in mammalian hosts and that the amino acid S42 of NS1 is critical for the virus to antagonize host cell interferon induction [11]. The specific exchange of E for D at position 92 of the A/HK/156/97 (H5N1) NS1 gene results in an order of magnitude increase in the quantum yield of IFN [12]. The presence of an alanine (A) residue at position 149 of the GS/GD/1/96(H5N1) NS1 protein antagonizes the induction of IFN protein levels in chicken embryo fibroblasts (CEFs) [13]. Amino acid mutations at position 64 of H3N2 IAV NS1 gene decrease NS1-mediated general inhibition of host protein synthesis and the interaction of the protein with CPSF30, leading the augmented IFN sensitivity and virus attenuation in mice [14]. All of these findings reveal the important role of NS1 protein and influenza viruses have evolved ways to counteract the host antiviral response.

The ability of host cells to recognize viral pathogens relies on pattern recognition receptors (PRRs) which trigger an antiviral response. Retinoic acid-inducible gene I product (RIG-I) has been identified as one of the key cellular sensors of RNA viruses. Once activated, it binds to cytosolic double-stranded RNA and initiates a signaling cascade that depends on the E3 ligase activity of tripartite motif family 25 (TRIM25), resulting in IFN and inflammatory cytokine induction [15]. Goffic et al. demonstrated a dual pathway by which RIG-I (but not MDA-5) mediates both type I IFN dependent antiviral signaling and a proinflammatory response during IAV infection [16]. In addition, the IAV viral protein NS1 binds to RIG-I and inhibits downstream activation of IRF-3, preventing the transcriptional induction of IFN [17]. These findings likely reveal the roles of RIG-I and the interactions between RIG-I and viral protein of influenza viruses.

Protein phosphorylation, a kind of post-translational modification, commonly occurs on threonine (T), serine (S), or tyrosine (Y) residues, and it regulates distinct activities of multifunctional proteins [18]. During the process of influenza infection, phosphorylation of the NS1 protein occurs rapidly after translation in the cytoplasm [19]. Biochemical studies on the NS1 protein have shown that some phosphorylation sites are able to affect virus replication and disrupt the host innate immune system. Kathum reported that T49 of NS1 is a negative charge, as phosphorylation of NS1 at T49 abrogates its IFN-suppressive function, leading to an attenuated virus phenotype in vitro and in vivo [20]. According to Hale, substitution of A for T at T215 in the human IAV NS1 protein reduces early viral propagation, although it only slightly affected the host interferon responses [21]. However, to date, there is only scarce information on the phosphorylation sites of the NS1 protein of swine influenza virus (SIV) and how phosphorylation may control NS1 function.

Here, we bioinformatically analyzed potential phosphorylation sites of the SIV NS1 protein. Upon generating recombinant influenza A/swine/Shanghai/3/2014(H1N1) viruses that encode mutant NS1 proteins (Y73F and S83A), we found that dephosphorylation at positions 73 and 83 of the NS1 protein attenuated virus replication and diminished the capability of NS1 to antagonize IFN-β expression. The function of NS1 protein phosphorylation is complicated, and this work will help to elucidate how phosphorylation mutations influence the function of the NS1 protein.

## Materials and methods

### Cells, viruses and antibodies

The H1N1 SIV A/swine/Shanghai/3/2014(SH/2014) strain was isolated during routine surveillance and sequenced by our laboratory. Madin-Darby canine kidney (MDCK) cells and the human epithelial kidney cell line (293 T) were obtained from the American Type Culture Collection (ATCC, Manassas, VA, USA) and grown in Dulbecco’s modified Eagle’s medium (DMEM) (Gibco, Grand Island, NY, USA) supplemented with 10% fetal bovine serum (FBS) (Invitrogen, Carlsbad, CA, USA) at 37 °C and 5% CO_2_. Rabbit polyclonal antibodies against RIG-I and horseradish peroxidase (HRP) conjugated goat anti-rabbit or anti-mouse secondary antibodies were purchased from Cell Signaling Technology (Danvers, MA, USA). A mouse monoclonal antibody against β-actin was purchased from Sigma (St Louis, MI, USA). Alexa Fluor 488-labeled goat anti-mouse IgG was purchased from Beyotime Biotechnology (Nantong, China). A mouse polyclonal antibody against the NS1 protein was generously provided by Professor Ying Fang at Kansas State University, USA.

### Prediction of phosphorylation sites

The full-length sequences of 30 SIV NS1 genes from H1N1, H1N2 and H3N2 subtype strains were obtained from GenBank (https://www.ncbi.nlm.nih.gov/). The amino acid sequences were aligned using the MegAlign program with DNASTAR software (DNASTAR., Madison, WI, USA), and conserved S, T and Y phosphorylation sites were obtained. To exclude false negatives and false positives, we used two websites to predict the possible phosphorylation sites of the NS1 protein. We uploaded amino acid sequences of NS1 to NetPhos 3.1 Server (http://www.cbs.dtu.dk/services/NetPhos/) and Scansite Motif Scan (https://scansite4.mit.edu/4.0/) [22, 23]. Scansite Motif Scan uses a peptide library-based search algorithm, and the NetPhos 3.1 Server uses an artificial neural network that has been extensively used in biological sequence analysis [24, 25].

### Phos-tag SDS-PAGE for NS1 phosphorylation state analysis

The pCAGGS-(SH/2014)-NS1 and pCAGGS-(SH/2014)-NS1 mutants Y73F, S76A, S83A, S151A, S161A and S195A were constructed by inserting the corresponding cDNAs into the EcoI/NheI sites of the pCAGGS vector. Then, 293 T cells grown in six-well plates were cotransfected with 2 μg of NS1 plasmid by using Lipofectamine 2000 transfection reagent. At 48 h posttransfection, we collected the cells and analyzed the phosphorylation state of the different mutants via western blotting using Phos-tag SDS-PAGE. The gel consisted of a separating and a stacking gel containing 100 μM Phos-tag and 10 mM MnCl_2_, which were added to the separating gel before polymerization [26]. The gel was then soaked in buffer solution containing 1 mM EDTA to eliminate manganese ions after electrophoresis [25]. The phosphorylation states of NS1 and NS1 mutants were determined by a western blot assay incubated with primary antibody and appropriate secondary antibody.

### Construction of plasmids for virus rescue

The eight gene segments of SH/2014 were cloned into the vRNA-mRNA bidirectional transcription vector pBD to rescue SH/2014, as described previously [13]. Mutations were introduced into the plasmid pBD-NS using overlap-PCR to generate the mutant NS segments: pBD-NS Y73F and pBD-NS S83A. The NS genes of the recombinant viruses were sequenced with the Lasergene software package (DNASTAR Inc., Madison, WI) to verify that the generated mutations were as expected. The recombinant viruses rSIV, rSIV NS1 Y73F and rSIV NS1 S83A were generated by cotransfection of 293 T cells with eight reverse-genetic plasmids with or without the substitution plasmid pBD-NS. At 72 h posttransfection, the supernatants were collected and propagated in MDCK cells with serum-free DMEM containing 2 μg/ml TPCK-trypsin. The viruses were titrated on MDCK cells and expressed as tissue culture infective dose (TCID_50_) using the Reed–Münch method.

### Virus growth curves

To evaluate virus growth kinetics, confluent monolayers of MDCK cells were infected with rSIV or NS1-mutant viruses at a multiplicity of infection (MOI) of 0.001 and incubated at 37 °C in 5% CO_2_. At 1 h post infection (hpi), the virus suspensions were removed and replaced with serum-free DMEM containing 2 μg/ml TPCK-treated trypsin. Supernatants were collected at 12, 24, 48, and 72 hpi and titrated on MDCK cells.

### Immunofluorescence assay

Immunofluorescence analysis was processed as described previously [27]. MDCK cells grown on glass coverslips were infected with rSIV or NS1-mutant viruses at an MOI of 1. At 6 hpi, the cells were washed with PBS and fixed in 4% paraformaldehyde for 10 min. The cells were then permeabilized with 0.5% Triton X-100 for 10 min, blocked in PBS containing 5% BSA for 30 min and stained with mouse anti-NS1 polyclonal antibody for 1 h. After washing with PBS, the coverslips were incubated with the Alexa Fluor 488-labeled goat anti-mouse antibody. The cell nuclei were stained with 4′,6′-diamidino-2-phenylindole (DAPI) (Beyotime). Finally, the coverslips were washed with PBS and visualized under a florescence microscope (Carl Zeiss, Oberkochen, Germany) using a 400× plan objective.

### Quantitative real-time RT-PCR (qRT-PCR)

293 T cells were infected with wt or NS1-mutant viruses at an MOI of 1 for different times, and total RNA was extracted using TRIzol Reagent (Invitrogen) according to the manufacturer’s recommendations. qRT-PCR was performed using specific primers to measure the mRNA levels of IFN-β, ISG56 and RIG-I, and β–actin was used as a control. The sequences of the primers are listed in Table 1. The amount of target mRNAs is presented as the change (n-fold) in induction relative to the control, as determined using the comparative cycle threshold (CT) method [28]. All primer sequences were designed using Primer3 software and were synthesized by Sangon Biotech Company (Shanghai, China).

**Table 1:** Primers used in this study

Name	Primer nucleotide sequence (5^′^-3^′^)^a^
NS1-F	TATTCGTCTCAGGGAGCAAAAGCAGGGTG
NS1-R	ATATCGTCTCGTATTAGTAGAAACAAGGGTGTTTT
NS1–73-F	CTGGAAGAAGAGTTTGATGAATCACTTA
NS1–73-R	TAAGTGATTCATCAAACTCTTCTTCCAG
NS1–76-F	AGAGTACGATGAAACGCTTAAAATGAC
NS1–76-R	GTCATTTTAAGCGTTTCATCGTACTCT
NS1–83-F	ACTATCGCTGCTGTGCCTGCTTCATGC
NS1–83-R	GCATGAAGCAGGCACAGCAGCGATAGT
NS1–151-F	CTTAGAGCTTTCGCTGAAGAAGGGGCGA
NS1–151-R	TCGCCCCTTCTTCAGCGAAAGCTCTAAG
NS1–161-F	GCGAAATCGCTCCGTTACCTTCTC
NS1–161-R	AGAAGGTAACGGAGCGATTTCG
NS1–195-F	ACAGTTCGAGTCGCTGAAGCTCTACAGAG
NS1–195-R	CTCTGTAGAGCTTCAGCGACTCGAACTGT
IFN-β-F	GCTGGAATGAGACTATTGTTGAGA
IFN-β-R	CAGTTTCGGAGGTAACCTGTAAG
ISG56-F	GCCATTTTCTTTGCTTCCCCT
ISG56-R	TGCCCTTTTGTAGCCTCCTTG
β-actin-F	TGGGTCAGAAGGACTCCTATG
β-actin-R	CAGGCAGCTCATAGCTCTTCT

^a^ Nucleotides that have been changed are shown in underlined

### RNA interference in 293 T cells

RNA interference was used to knock down endogenous RIG-I. 293 T cells grown to 40 to 50% confluence in 6-well plates were transfected with 100 nmol of siRNA using 3 μl of Lipofectamine 2000 (Invitrogen) in Opti-MEM (Invitrogen). Twenty-four hours after transfection, the medium was removed. The cells were then infected with wt or mutant viruses at an MOI of 1 for 24 h and then harvested for analysis by western blot analysis or real-time PCR. Scrambled siRNA was used as a negative control. The siRNAs were designed and synthesized by Gene Pharma Company (Shanghai, China) with the following sequences: siRIG-I, 5′-GCCCAUUGAAACCAAGAAAUU-3′; nontargeting control siRNA, 5′-UUCUCCGAACGUGUCACGUTT-3’ [29].

### Statistical analysis

The data are expressed as the means ± standard deviations (SD). The significance was determined with a two-tailed independent Student’s t-test. A *p*-value of < 0.05 was considered to be statistically significant.

### Results

#### Prediction of phosphorylation sites in the SIV NS1 protein

To identify possible NS1 phosphorylation sites, the NetPhos 3.1 and Kinase Scansite 4.0 databases were used. As shown in Table 2, 18 phosphorylation sites were bioinformatically predicted. On our conjecture, phosphorylation sites with significant biological functions should be highly conserved and should exist in all SIV subtypes. We checked 18 predicted sites in 30 SIV NS1 genes to exclude strain-specific sites, covering H1N1, H1N2 and H3N2 subtypes. Of these sites, we selected conserved sites, including 6 S sites, 1 T site and 1 Y site for site-directed mutagenesis. Aside from the sites that are reported, six sites, Y73, S76, S83, T151, S161, and S195, were subjected to further investigation.

**Table 2:** Summary of predicted phosphorylation sites

Conservation	site^a^	Amino acid^b^	Netphos3.1 value^c^	Scansite value^d^
Conserved	42	S	0.98	0.881
	49	T	0.6	0.784
	73	Y	0.953	0.745
	76	S	0.856	0.727
	83	S	0.785	0.713
	151	S	0.737	0.777
	161	S	0.572	0.7
	195	S	0.891	0.687
Non-conserved	47	S	0.84	0.599
	48	S	0.943	0.626
	87	S	0.863	0.63
	91	T	0.585	0.609
	94	T	0.517	0.57
	99	S	0.504	0.589
	114	S	0.584	0.608
	129	T	0.866	0.896
	143	T	0.665	0.715
	205	S	0.649	0.714

^a^ Phosphorylation sites were predicted for NS1 protein of SIV strain SH/2014

^b^ S is for Serine, T is for Threonine and Y for Tyrosine predictions

^c, d^ Predicted sites by NetPhos3.1 or Scansite with a score of > 0.5

### Phosphorylation analysis of the predicted phosphorylation sites

**Fig. 1:**
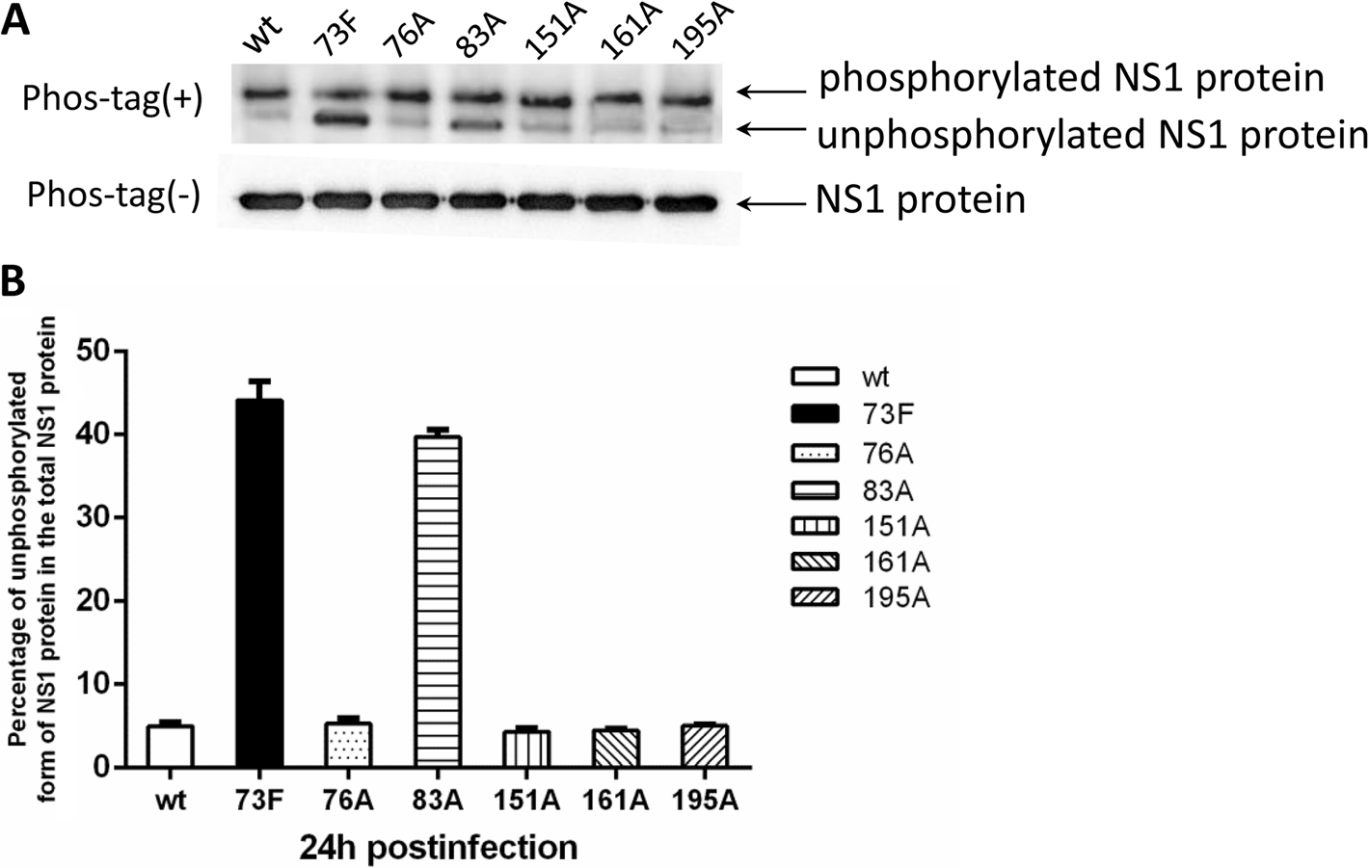
Phosphorylation status analysis of predicted NS1 protein phosphorylation sites in 293 T cells. **a** 293 T cells transfected with plasmid pCAGGS-NS1 or pCAGGS-NS1 mutant Y73F, S76A, S83A, T151A, S161A, and S195A were lysed 48 h after transfection. Protein samples were separated by SDS-PAGE containing Phos-tag (designated as Phos-tag(+))and general SDS-PAGE (designated as Phos-tag(−)),and analyzed by western blotting with mouse anti-NS1 antibodies. The Phos-tag is a ligand that shifts the mobility of phosphorylated NS1proteins.The bottom band is the NS1 protein in its unphosphorylated form. **b** The percentage of unphosphorylated NS1 protein in the total NS1 protein. The intensities of the unphosphorylated NS1 and total NS1 protein bands were quantified using ImageJ software. The ratios of unphosphorylated NS1 to total NS1 protein are shown

293 T cells were transfected with the different NS1 variants and we assayed the level of NS1 phosphorylation by western blotting using SDS-PAGE gels containing Phos-tag, a ligand that shifts the mobility of phosphorylated proteins. After electrophoresis, NS1 proteins could be separated into several bands due to the decreased mobility of phosphorylated NS1 proteins. As shown in Fig. 1, unphosphorylated NS1 protein was difficult to detect in cells transfected with pCAGGS-NS1 expressing wt NS1 proteins at 48 hpi. However, the amount of unphosphorylated NS1 protein increased and when the amino acid at position 73 was mutated to F and when the amino acid at position 83 was mutated to A, suggesting that the NS1 proteins were phosphorylated at positions 73 and 83.

### Dephosphorylation of the NS1 protein at position 73 and 83 affects SIV replication

**Fig. 2:**
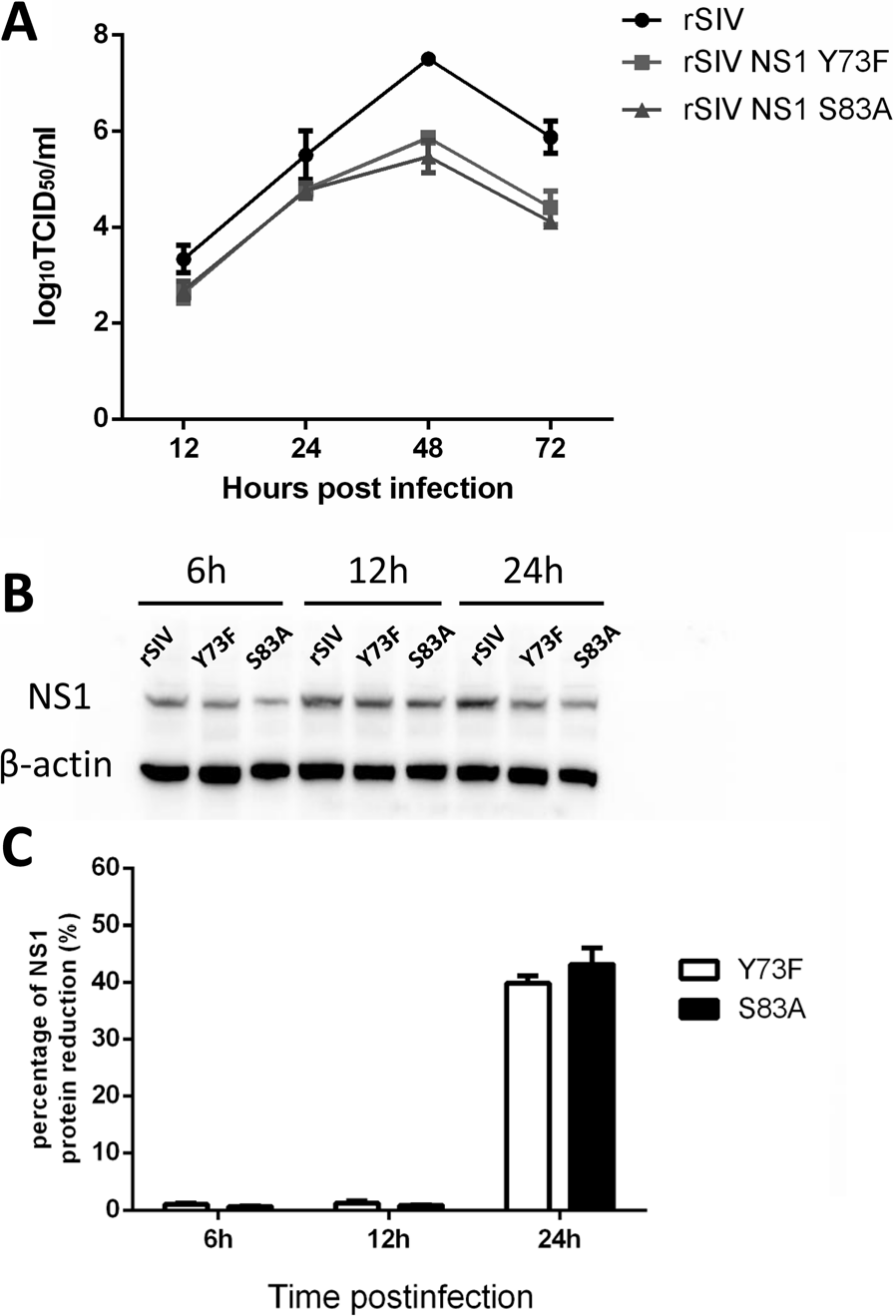
Growth kinetics of recombinant viruses with NS1 mutants lacking a phosphorylation site. **a** Growth kinetics of the recombinant viruses in MDCK cells. Recombinant viruses possessing wild-type NS1, NS1 Y73F and NS1 S83A were generated by reverse genetics, as described in the Materials and Methods. MDCK cells were infected with rSIV or mutated virus (rSIV NS1 Y73F and rSIV NS1 S83A) at an MOI of 0.001. Supernatants were collected at 12, 24, 48, and 72 hpi, and virus titers were determined by TCID_50_ assay. The mean values from three independent experiments are shown for each sample. **b** Expression kinetics of NS1 protein during infection. MDCK cells on 6-well plates were infected at an MOI of 0.001 with rSIV or mutated viruses, and cell lysates were collected at the indicated time points for western blot analysis of NS1 protein levels.C. Percentage of the NS1 protein reduction level. The percentage of protein reduction (%) was calculated by the formula [(NS1 expression of rSIV infected cells - NS1 expression of mutant viruses infected cells)/ NS1 expression of rSIV infected cells] × 100%; Abscissa: time points after infection

Recombinant viruses encoding NS1 with Y-to-F substitution at position 73 and S-to-A substitution at position 83 were generated and denoted the rSIV NS1 Y73F and rSIV NS1 S83A viruses, respectively. To measure the growth rates of the two mutant viruses, MDCK cells were infected with wt or NS1-mutant viruses at an MOI of 0.001, and supernatants were analyzed for virus progeny by TCID_50_ assay at the indicated time points. The result showed that two mutant viruses grew similarly in MDCK cells, and their titers peaked at 48 h, with values of 5.87 and 5.47 TICD_50_/ml, respectively (Fig. 2a). The titers of each mutant virus were significantly lower than those of the wild-type virus at 48 and 72 hpi. To investigate whether the growth defects were correlated with any deficiencies in viral protein expression, we also analyzed the NS1 expression level of NS1 Y73F and NS1 S83A mutants in virus-infected cells by western blotting. As shown in Fig. 2b, the protein expression levels of NS1 in the wt virus and the two mutated viruses were similar at 6 hpi and 12 hpi. However, at 24 hpi, the NS1 protein expression was reduced dramatically in the cells infected with the NS1 Y73F and NS1 S83A mutant viruses compared with those infected with the wt virus, showing reductions of approximately 40% (Y73F) and 44% (S83A). The band intensities were conducted with ImageJ software and the percentage of NS1 protein reduction (%) is shown in Fig. 2c. Collectively, the findings indicated that dephosphorylation of the H1N1 SIV NS1 protein at position 73 and 83 suppressed protein synthesis and recombinant virus assembly.

### Viruses with NS1 Y73F and NS1 S83A dephosphorylation show similar subcellular localization

**Fig. 3:**
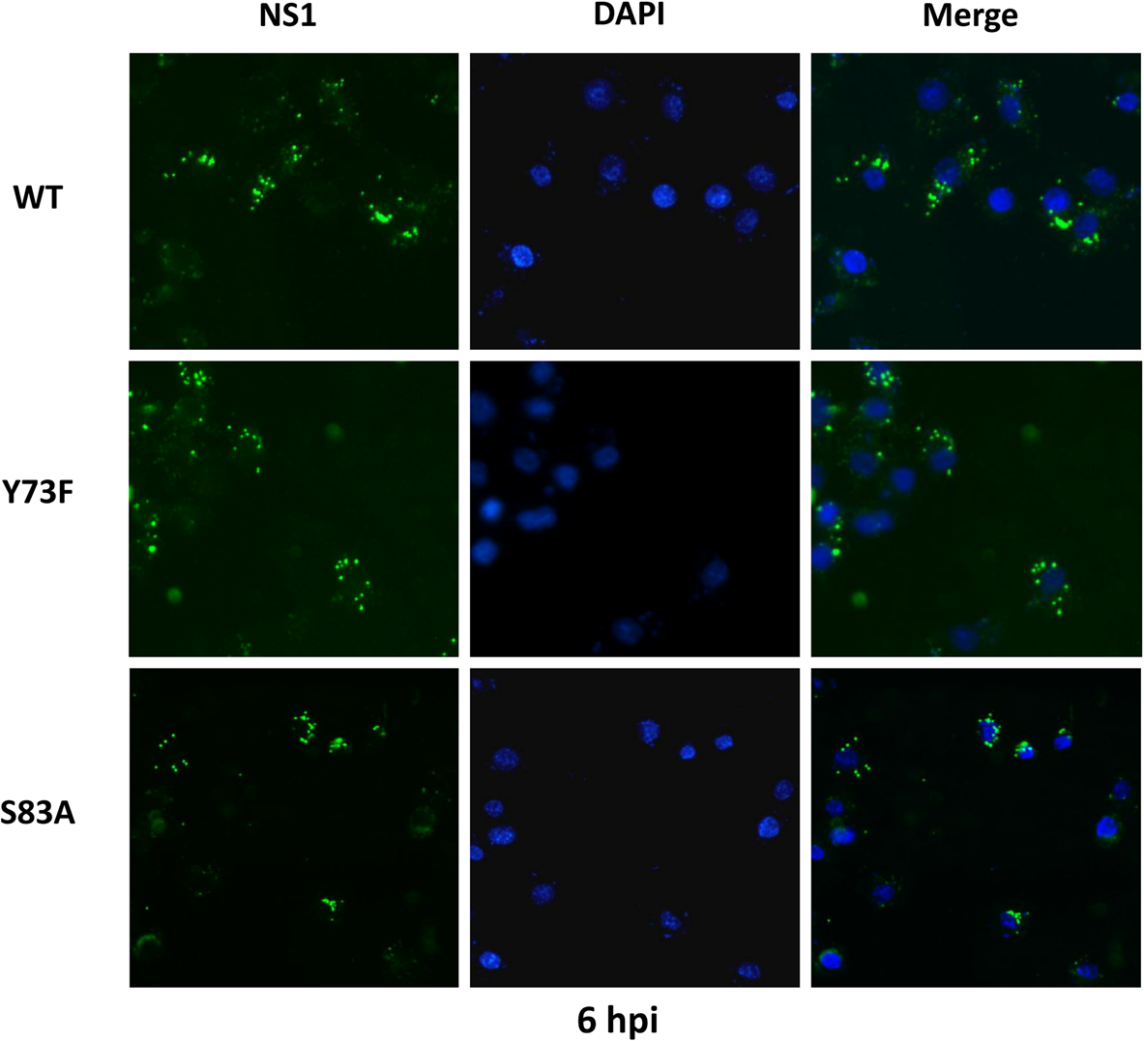
NS1 protein localization is not affected by Y73F or S83A dephosphorylation. MDCK cells were infected with rSIV and mutated viruses at an MOI of 1. At 6 hpi, the cells were fixed and stained with a specific anti-NS1 polyclonal antibody and a Alexa Fluor 488–labeled secondary antibody (green). Nuclei were stained with DAPI (blue). The localizations of NS1 proteins were visualized using a Carl Zeiss fluorescence microscope. Merge: merged image of double immunofluorescence staining of the same field

To determine whether dephosphorylation of the NS1 protein at position 73 and 83 disrupted NS1 nuclear localization, MDCK cells were infected at an MOI of 1 for 6 h and then examined by immunofluorescence microscopy. As shown in Fig. 3, no clear change in nuclear localization was observed at 6 hpi. Most of the mutants and wt virus NS1 proteins accumulated in the cytoplasm, and little was detected in the nucleus. Overall, the subcellular localization of the NS1 Y73F and the NS1 S83A mutant proteins was very similar to that of the wt NS1 protein; thus, these point mutations are insufficient to alter nuclear localization.

### Effect of NS1 changes on the type I IFN response

**Fig. 4:**
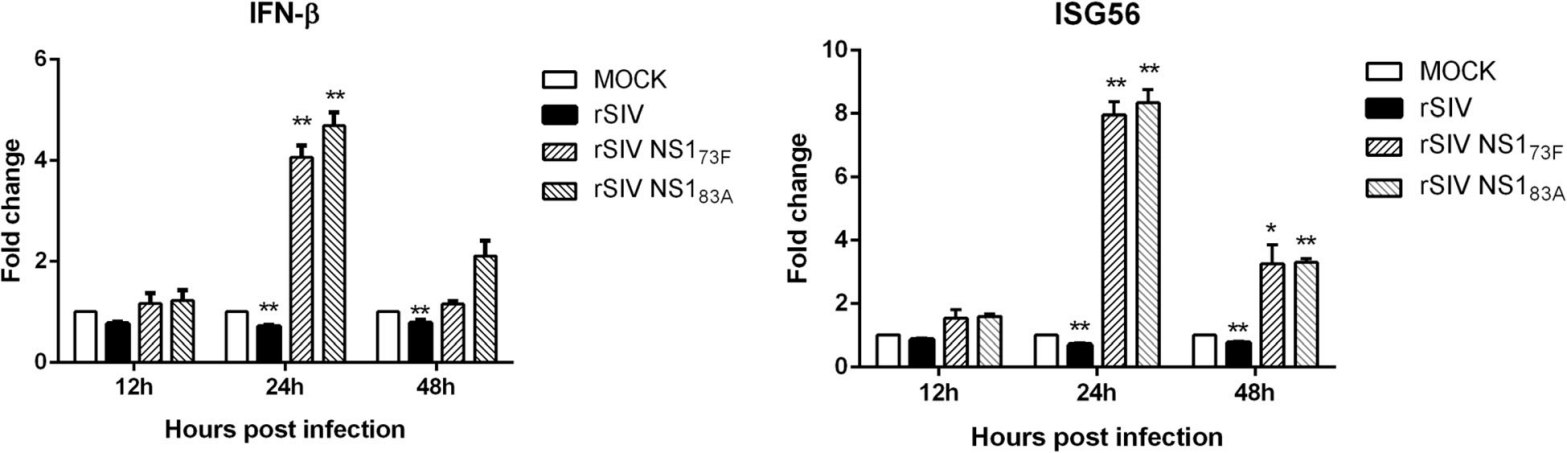
Y73F and S83A substitution affects IFN-β antagonistic properties of NS1. **a**, **b** 293 T cells were infected with rSIV or mutated virus at an MOI of 1 for different times. IFN-β and ISG56 mRNA levels in the cell lysates were quantified by real-time PCR. Values represent n-fold expression of MOCK-infected cells and are displayed as the means ± SDs of three independently repeated experiments. “MOCK” represents samples from uninfected control cells

As the NS1 protein is thought to be a key determinant of IFN antagonism, we wondered whether the replication attenuated caused by dephosphorylation at Y73 and S83 might be due to increased induction of IFN-β. Therefore, 293 T cells were infected with SH/2014 recombinant virus containing wt NS1 or mutant NS1 for 12, 24, and 48 h, and the levels of IFN-β and ISG56 mRNA were investigated by real-time PCR. As shown in Fig. 4, wt NS1 virus infection led to a weak ability to induce IFN-β compared to Mock cells. However, infection with the rSIV NS1 Y73F or S83A virus induced significantly higher levels of IFN-β expression (4.06-fold and 4.49-fold, respectively) at 24 h after infection. Likewise, the expression of ISG56 upregulated by approximately 7.96-fold (rSIV NS1 Y73F virus) and 8.34-fold (rSIV NS1 S83A virus) compared to that in MDCK cells at 24 hpi. These data demonstrated that phosphorylation at positions 73 and 83 was critical for the NS1 protein to inhibit IFN expression.

### RIG-I contributes to IFN-β production during infection with the Y73F and S83A mutants

**Fig. 5:**
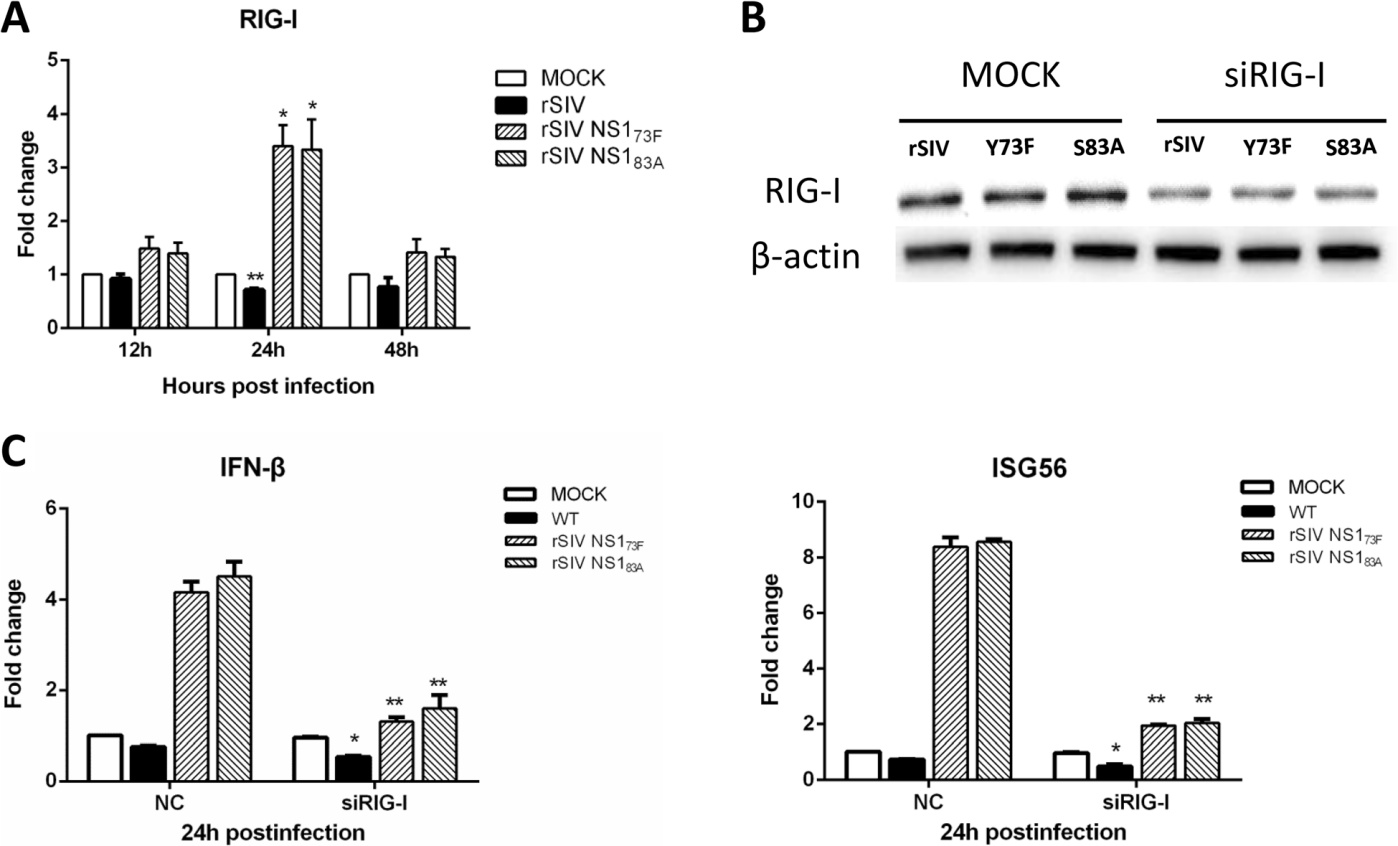
RIG-I contributes to IFN-β production during infection with Y73F and S83A mutants. **a** 293 T cells were infected with rSIV or mutated virus at an MOI of 1 for 24 h, and the levels of RIG-I mRNA were investigated by real-time PCR. **b** 293 T cells were transfected with siRNA specific for RIG-I (100 nmol/ml) or control scrambled siRNA for 48 h and then inoculated with rSIV or mutated virus at an MOI of 1. The cells were harvested at 24 hpi. The protein levels were determined using an anti-RIG-I antibody, and β-actin was used as the protein loading control. **c** 293 T cells transfected with RIG-I-specific siRNA were infected with rSIV or mutated viruses at an MOI of 1 for 24 h and then harvested for determination of IFN-β and ISG56 mRNA levels by real-time PCR

To reveal whether changes in the type I IFN response caused by dephosphorylation at positions 73 and 83 were mediated by RIG-I, we infected 293 T cells with the 2 mutant viruses and the wt virus at an MOI of 1 and measured RIG-I expression at 24 hpi. As shown in Fig. 5a, recombinant wt virus infection exerted an inhibitory effect on RIG-I transcription; however, over 3-fold greater RIG-I expression was observed in Y73F and S83A mutant infected cells then in control cells at 24 hpi. To further confirm our observation that RIG-I mediates IFN-β production during infection by NS1 Y73F and S83A mutants, we collected RIG-I knockdown cells and subjected them to real-time PCR for IFN-β and ISG56 quantification. 293 T cells were transfected with RIG-I-specific siRNA, and a scrambled, nonspecific siRNA was utilized as a control. After cells were infectd with the two mutant viruses and the wt virus for up to 24 h, the western blotting results showed that RIG-I expression was approximately 50% lower in cells transfected with RIG-I-specific siRNA than with control siRNA. In addition, after transfecting the mutant virus– and wt virus–infected cells with RIG-I-specific siRNA, we observed that RIG-I interference inhibited the induction of IFN-β and ISG56 in the wt virus-infected cells. Furthermore, RIG-I interference dramatically impaired the induction of IFN-β and ISG56 in NS1 Y73F and S83A mutant-infected cells. Taken together, these data indicated that dephosphorylation at positions 73 and 83 of the NS1 protein affected the antiviral state in the host cells, partly through the RIG-I pathway.

## Discussion

Reversible phosphorylation, a form of protein modification, is a molecular switch that has important effects on protein functions associated with virus/host interactions. Phosphorylation of the IAV viral proteins has been demonstrated, and the phosphorylation sites have also been found to be highly conserved [30]. Phosphorylation of conserved S residues of NEP has been shown to affect the nuclear export activity or polymerase activity-enhancing function [31]. Phosphorylation sites at the S165 IVA NP protein are involved in self-oligomerization, vRNP activity, and subsequent effects on viral growth [32]. Y 132 phosphorylation of the IVA M1 protein is crucial for virus replication by controlling the nuclear import of M1 [33]. Multiple sites in NS1 have also been reported and appear to affect the protein’s activity [19, 20]. The function of viral protein phosphorylation is complicated but is involved in the process of virus reproduction.

In this study, 18 candidate phosphorylated amino acid residues of the NS1 protein were predicted through bioinformatics analysis, and we analyzed the phosphorylation statuses of the conserved sites through Phos-tag SDS-PAGE. In this technique, phosphorylated proteins can be visualized as slower-migrating bands than the corresponding dephosphorylated protein bands, as the Phos-tag is a selective phosphate-binding tag molecule. As shown in Fig. 2, 293 T cells were transfected with six NS1 mutant-expressing plasmids to examine their phosphorylation status by Phos-tag SDS-PAGE assay. We found that Y-to-F substitution at position 73 or S-to-A substitution at position 83 increased the levels of unphosphorylated NS1 protein, suggesting that Y73 and S83 are phosphorylated in wt NS1 proteins. The NS1 protein has an average length of 230 amino acids which is notionally divided into two distinct functional domains. Y73 is located in the N-terminal RNA-binding domain (residues 1–73), and mutations at this site might affect the binding activity to dsRNA, an intermediate in the process of virus replication. In contrast, S83 lies in the C-terminal ‘effector’ domain (residues 74–230). Mutations at this site might affect its interactions with host-cell proteins. The NS1 protein may exist in the form of homodimer and both the RNA binding and effector domains are involved in its stability and polymerization status [34].

In this study, we rescued a classic swine influenza virus (A/swine/Shanghai/3/2014(H1N1)) and its NS1 protein mutants via reverse genetics and compared their replication ability in cultured cells. The growth curves revealed that dephosphorylation of Y73 and S83 caused a reduction in the amount of SIV particles in the cell culture supernatant, as well as viral protein synthesis, compared to wild-type virus at the same time point after infection. It has been shown earlier that in human IAV, phosphorylation of S42 and T49 attenuated virus replication, while T215 phosphorylation does not affect virus replication [35]. We hypothesize that phosphorylation of the NS1 protein can both up- and downregulate replication of SIV and that such differences may depend on the phosphorylated sites, which also differ among different types of influenza viruses. In the present study, it is possible that the efficient generation of H1N1 SIV required the presence of phosphorylated Y at position 73 and S at position 83 of the encoded NS1 protein.

It is well known that type I IFN antagonism is one of the main biological properties of NS1 protein on host cells. Therefore, we explored whether the attenuated replication caused by dephosphorylation at Y73 and S83 in NS1 of the SH/2014 virus might be due to elevated induction of IFN-β. Indeed, both of the NS1 mutants resulted in strong IFN gene induction, indicating that dephosphorylation at Y73 and S83 is less capable of suppressing the cellular IFN response. RIG-I has been identified as a cellular sensor of RNA virus infection resulting in type I IFN induction, and IAV can inhibit type I IFN production through its NS1 protein by inhibiting RIG-I [36, 37]. In this study, we found that knockdown of RIG-I dramatically impaired the induction of IFN-β and ISG56 in NS1 Y73F or S83A mutant-infected cells, indicating that RIG-I plays a role in the IFN-β response upon rSIV NS1 Y73F and rSIV NS1 S83A infection.

## Conclusions

In conclusion, we first identified two functional phosphorylation sites in the H1N1 SIV protein: Y73 and S83. We found that dephosphorylation at positions 73 and 83 of the NS1 protein attenuated virus replication and reduced the ability of NS1 to antagonize IFN-β expression. The present study helps to clarify the mechanism by which NS1 protein phosphorylation affects SIV propagation and interferon-antagonistic activity.

## Data Availability

All data generated or analysed during this study were available from the corresponding author on reasonable request.

## Abbreviations

293 T: Human epithelial kidney cell line
DMEM: Dulbecco’s modified Eagle’s medium
hpi: Hours post infection
IAV: Influenza A virus
MDCK: Madin-Darby canine kidney cells
NS1 protein: Nonstructuralprotein 1
RIG-I: Retinoic acid-inducible gene1
S: Serine
SIV: Swine influenza virus
T: Threonine
TCID_50_: 50% Tissue culture infective dose
TRIM25: Tripartite motif family 25
Wt: Wild-type
Y: Tyrosine
